# News and Views on STAT3 Psychopathology

**DOI:** 10.3389/fgene.2021.791201

**Published:** 2021-11-26

**Authors:** Sara H. Sadok, Rayssa L. Borges-Medeiros, Paula V. B. Macêdo, João Ricardo M. de Oliveira, High IgE Consortium

**Affiliations:** ^1^ Keizo Asami Laboratory, Federal University of Pernambuco, Recife, Brazil; ^2^ Neuropsychiatric Department, Federal University of Pernambuco, Recife, Brazil

**Keywords:** Job's syndrome, high IgE, signal transducer and activator of transcription, neuropsychiatric disorders, biomarker

## Introduction

The work of Reisinger and others from Austria, despite being based on experiments conducted on rodents, claims an old ambition of neuroimmunologists, namely, to integrate the main psychopathology disturbances into a unified paradigm (Reisinger et al., 2020). These authors published in the Molecular Psychiatry Journal a study concerning the signal transducer and activator of transcription 3 protein (STAT3) and its regulation of emotional behavior in knockout and knockdown *Stat3* mice (Reisinger et al., 2020). They highlighted the potential role played by Stat3 protein in the serotonergic system and, therefore, suggested the implications in the genesis of neuropsychiatric disorders, reinforcing the well-known immune hypothesis of mental illness.

By analyzing the achieved results, it is essential to rethink the current paradigm of human neuropsychiatry disorders concerning diagnosis and consider that biological markers might be a breakthrough. However, the main challenge still is to tackle the first domino piece on the neuroimmune cascade leading to sustained changes triggered by environment distress of even “primary” or “endogenous” and sometimes even predictable swings on mood, attention, memory and full vigilance. Curiously, the neuroimmune concept is being explored for over a century and we remember the 1927 Nobel Winner, Julius Wagner-Jauregg, also from Austria, and his work using many microbes to treat mainly psychotic and catatonic patients.

## Discussion

It is important to point that current database, such as the Allen Brain Atlas (^©^ 2008 Allen Institute for Brain Science. Allen Developing Mouse Brain Atlas. Available from: https://developingmouse.brain-map.org/, and ^©^ 2004 Allen Institute for Brain Science Allen Mouse Brain Atlas. Available from: https://mouse.brain-map.org/) and the mouse brain vasculature map provided by Betsholtz Lab database (Vanlandewijck et al., 2018), in wild-type mice shows that *Stat3* is widely expressed in different cell types, showing peaks of expression in arterial smooth muscle cells (aSMC) and subtype 2 vascular fibroblast-like cells (Fb2), and a lower level at astrocytes (AC). In the developing mouse brain, *Stat3* has a similar expression pattern in all areas. However, there is a change in the expression pattern between the embryonic days and postnatal age in the developing mouse brain, since during the postnatal days (P4, P14 and P28) the *Stat3* expression values increase. In the adult mouse brain, most areas also share a similar expression profile of this gene.

In the context of the classical monoamine hypothesis, for most part of the time cerebrospinal fluid levels of serotonin, dopamine and noradrenaline and their respective enzymes, as well as other neurotransmitters, have been considered as the main biomarkers of mental illnesses, especially in major depressive disorders. Recently, studies have also suggested that neurotrophic and inflammatory biomarkers may play a role in the pathogenesis of these psychiatric disorders. There is increased evidence that elevated levels of cytokines, C-reactive protein and interleukins are related to the pathogenesis of psychiatric conditions and hence could be used as measurable indicators in the scenario of mental disorders (Lozupone et al., 2019). Moreover, the potential use of biomarkers is not limited to aid with precision regarding diagnostic criteria, but also related to response to treatment, stratification of patients and appropriate treatment choice in the era of Personalized Medicine (Garcia-Gutiérrez et al., 2020).

In humans, *STAT3* gene variants, associated with both autosomal dominant and sporadic form are linked to the Hyper Immunoglobulin E (Hyper-IgE) syndrome (also known as Job’s disease). The Hyper-IgE syndrome is a group of different inborn errors of immunity marked by the presence of recurrent infections, multisystem organs involvement, characteristic facial dysmorphism, dermatitis, vasculopathy, and extremely elevated serum levels of IgE (Tsilifis et al., 2021). Although *STAT3* variants in animal models were involved in behavioral implications, also mentioned by Reisinger et al., psychiatric symptoms were not listed in the clinical description of the referred syndrome.

It has come to our attention that similar, but mild, symptomatology of this syndrome might be present in some patients with mildly increased IgE serum levels, which remains not documented in the literature (Sadok et al., 2021). As a consequence, these changes might result in a broad range of clinical presentations, involving moderate symptoms and including neuropsychiatric disorders. The referred manifestations are usually considered benign and common in the population, depending on the type of complication and the system affected. In addition, they are intensified on consanguineous families (Otto et al., 2020).

During the COVID-19 pandemic, the neurological implications of the infection and the affinity of the virus for the brain grabbed the attention of the scientific community. In addition, the relation between neuropsychiatric symptoms development on COVID-19 patients and increased pro-inflammatory cytokines response to the virus infection has been demonstrated (Varatharaj et al., 2020). Therefore, this information reinforces the relevance of biomarkers screening for risk stratification or even diagnosis. Interestingly, Troyer et al. (2020) detected on fatal COVID-19 patients, increased pro-inflammatory interleukin 6, one of the triggers for STAT3 pathway activation.

## Conclusion

Thus, we believe that the findings described by the authors in the manuscript, along with our hypothesis of neuropsychiatric involvement of STAT3 Hyper-IgE patients and the importance of biomarkers, should be considered in future research.

## References

[B1] García-Gutiérrez M. S. Navarrete F. Sala F. Gasparyan A. Austrich-Olivares A. Manzanares J. (2020). Biomarkers in Psychiatry: Concept, Definition, Types and Relevance to the Clinical Reality. Front. Psychiatry. 11, 432. 10.3389/fpsyt.2020.00432 32499729 PMC7243207

[B2] Lozupone M. La Montagna M. D’Urso F. Daniele A. Greco A. Seripa D. (2019). The Role of Biomarkers in Psychiatry. Adv. Exp. Med. Biol. 1118, 135–162. 10.1007/978-3-030-05542-4_7 30747421

[B3] Otto P. A. Lemes R. B. Farias A. A. Weller M. Lima S. O. A. Albino V. A. (2020). The Structure of First-Cousin Marriages in Brazil. Sci. Rep. 10 (1), 15573. 10.1038/s41598-020-72366-z 32968083 PMC7511957

[B4] Reisinger S. N. Sideromenos S. Horvath O. Derdak S. Cicvaric A. Monje F. J. (2020). STAT3 in the Dorsal Raphe Gates Behavioural Reactivity and Regulates Gene Networks Associated With Psychopathology. Mol. Psychiatry. 26, 2886–2899. 10.1038/s41380-020-00904-2 33046834 PMC8505245

[B5] Sadok S. H. Borges-Medeiros R. L. de Oliveira J. R. M. (2021). Editorial of Concern. J. Mol. Neurosci., 1. 10.1007/s12031-021-01904-9 PMC8415922 34480319

[B6] Troyer E. A. Kohn J. N. Hong S. (2020). Are We Facing a Crashing Wave of Neuropsychiatric Sequelae of COVID-19? Neuropsychiatric Symptoms and Potential Immunologic Mechanisms. Brain Behav. Immun. 87, 34–39. 10.1016/j.bbi.2020.04.027 32298803 PMC7152874

[B7] Tsilifis C. Freeman A. F. Gennery A. R. (2021). STAT3 Hyper-IgE Syndrome-An Update and Unanswered Questions. J. Clin. Immunol. 41 (5), 864–880. 10.1007/s10875-021-01051-1 33932191 PMC8249299

[B8] Vanlandewijck M. He L. Mäe M. A. Andrae J. Ando K. Del Gaudio F. (2018). A Molecular Atlas of Cell Types and Zonation in the Brain Vasculature. Nature. 554, 475–480. 10.1038/nature25739 29443965

[B9] Varatharaj A. Thomas N. Ellul M. A. Davies N. W. S. Pollak T. A. Tenorio E. L. (2020). Neurological and Neuropsychiatric Complications of COVID-19 in 153 Patients: a UK-Wide Surveillance Study. Lancet Psychiatry. 7 (10), 875–882. 10.1016/S2215-0366(20)30287-X 32593341 PMC7316461

